# Anti-Shigellosis Activity of *Cola anomala* Water/Ethanol Pods Extract on *Shigella flexneri-*Induced Diarrhea in Rats

**DOI:** 10.1155/2019/6706230

**Published:** 2019-11-11

**Authors:** Henri Wambe, Paul Aimé Noubissi, Michel Archange Fokam Tagne, Angèle Foyet Fondjo, Gaëtan Olivier Fankem, Idrice Kamtchouing, Joseph Ngakou Mukam, Télesphore Bénoît Nguelefack, René Kamgang

**Affiliations:** ^1^Department of Biology, Faculty of Science, University of Dschang, P.O. Box 67, Dschang, Cameroon; ^2^Department of Zoology and Animal Physiology, Faculty of Science, University of Buea, P.O. Box 63, Buea, Cameroon; ^3^Department of Biological Science, Faculty of Science, University of Ngaoundéré, P.O. Box 454, Ngaoundéré, Cameroon; ^4^Department of Applied Sciences for Health, Higher Institute of Applied Sciences, University Institute of Gulf of Guinea, P.O. Box 12489, Douala, Cameroon; ^5^Animal Physiology Laboratory, Faculty of Science, University of Yaoundé I, P.O. Box 812, Yaoundé, Cameroon; ^6^Laboratory of Endocrinology and Radioisotopes, Institute of Medical Research and Medicinal Plants Studies (IMPM), Yaoundé, Cameroon

## Abstract

This study was undertaken to evaluate the activities of water/ethanol *Cola anomala* pods extract. *In vitro* antimicrobial susceptibility was determined by the disk diffusion method; the minimum inhibitory concentration and minimum bactericidal concentration were determined by agar dilution technique. *In vivo*, shigellosis was induced in healthy Wistar albino rats by oral administration of *Shigella flexneri* inoculum, 12 × 10^8^ CFU/mL. At the onset of diarrhea, infected and normal control animals were subdivided into various groups treated with distilled water, with water/ethanol *Cola anomala* pods extract at 25, 50, or 100 mg/kg, or with ciprofloxacin, 2.5 mg/kg. After one-week treatment, rats were sacrificed, and blood and colon were collected. Blood was used for blood cell count. A portion of the colon served for histological studies while homogenate from the remaining part was centrifuged and the supernatant was collected for the determination of NO, PGE_2_, IL-1*β*, and TNF-*α* levels. *In vitro*, water/ethanol *Cola anomala* pods extract showed to be bactericidal, with a minimum inhibitory concentration of 2.0 mg/mL and a minimum bactericidal concentration of 3.0 mg/mL. In diarrheic rats, the extract significantly (*P* < 0.01) increased the white blood cells and significantly (*P* < 0.01) decreased stool *Shigella* density from the first to the seventh day of treatment. It partially restored the structure of eroded intestine epithelium and prevented weight loss; the dose dependently and significantly (*P* < 0.001) decreased NO, IL-1*β*, and TNF-*α* production in the colon and was found to have no significant effect on PGE_2_ production. These results support the use of this plant in traditional medicine in the treatment of gastrointestinal ailments.

## 1. Introduction

Diarrheal diseases result from an intestinal transit disorder characterized by loose or liquid stools, in abnormally high amounts or with an increased occurrence frequency, about three times a day during few days to few weeks depriving the body from its necessary mineral salts [1]. Diarrhea sometimes results from an increased motility and secretion of the digestive canal or decreased fluid absorption, leading to water and electrolytes (Na^+^, Cl^−^, and K^+^) loss [2]. Usually, they can be a symptom of an intestinal infection caused by various microorganisms: parasites, bacteria, or viruses [2], and they remain the second main cause of death in infant not up to five, while accounting for nearly 1.7 billion cases with 525,000 deaths every year [3]. The probability of diarrhea occurrence is about 39.1% for Sub-Sahelian developing African countries, compared to 7.2% in developed countries [4].

In Cameroon, among the major causes of child mortality, diarrhea comes first. And among the leading causes of morbidity, they rank third with a prevalence of 13.6% nationwide. So, by their epidemic-endemic nature, diarrheal diseases constitute a major threat for the country [5]. Among the most dangerous diarrhea cases in humans is the bacillary dysentery or shigellosis, which has been responsible for major epidemics that have become historical in the world [6]. Shigellosis is an acute invasive intestinal infection caused by bacteria of the genus *Shigella*; it is endemic in most developing countries, being the leading cause of bloody diarrhea worldwide with at least 80 million cases of bloody diarrhea and 700,000 deaths each year [7]. Shigellosis is responsible for about 22% of deaths from diarrhea per year worldwide [8]. In Cameroon, shigellosis accounts for about 4.5% of diarrhea cases per year [4]. In 2010, the episode which occurred in the City of Buea had an antibiotic resistance rate of 90% [9].

Among *Shigella* species, *Shigella dysenteriae* type 1 (Sd1) is responsible for the most dangerous form of the disease and can cause large epidemics on a regional scale. *Shigella flexneri* is the main cause of endemic shigellosis in developing countries [10]. The main obstacle to shigellosis control is the high rate with which *Shigella* is transmitted from person to person and the rate of occurrence of resistance of the bacteria to antibiotics [10]. Treatment of shigellosis is based on the administration of an anti-infectious agent accompanied by rehydration and supplementation of the zinc [10]. Zinc reduces the secretion of chloride ions by inhibiting the potassium canals of the basolateral membrane, on the one hand, and improves the absorption capacity of water and electrolytes and strengthens the intestinal immune response on the other hand. Ciprofloxacin, mostly used as the main antishigellosis drug, showed to be the cause of arthropathy in children. The use of these antibiotics and other antidiarrheal is currently challenged by the cost as well as the availability of these synthesized molecules and by the development of multiresistant microbial strains [2]. This, thus, confirms the need of an alternative medicine promisingly from medicinal plants locally available for the treatment of these diseases [11, 12].

Cola is a tropical African tree belonging to the Sterculiaceae family. The genus is made up of about 140 species, with the most commonly exploited being *Cola acuminata*, *Cola nitida*, and *Cola anomala* [13]. Cola nut, the mature fruits of the *Cola* sp., is a very important aspect of the tradition in Cameroon, as well as for medicinal purposes. Various medicinal and pharmacological values have been observed in species of *Cola*. Cola nuts are sometimes used against whooping cough and asthma [14], to treat malaria, nervous debility, weakness, lack of emotion, nervous diarrhea, depression, anxiety, fever, and as antimicrobial agents [15]. It is also used to increase physical capacity for endurance during fatigue and to stimulate weak heart [15]. Cola nut has a bitter taste and a great caffeine content [15]; the ingested fruit acts as stimulants creating an euphoric state [15]. Phytochemical screening revealed the presence of tannins, anthraquinones, alkaloids, saponins, and cardenolides in *Cola* species [14, 15].

Traditionally, the fruit follicles, the bark, and leaves of *Cola anomala* are used by the population from the Western Region of Cameroon as a remedy against dysentery, coughs, diarrhea, and vomiting. The prior reports of antibacterial and antiparasitic activities [14] support the use of *Cola* fruit to treat diarrhea and other gastrointestinal infections. It is therefore necessary to establish scientific evidence for therapeutic use of *Cola anomala*, as it may potentially be a useful source of new lead compounds to be an input for the drug development process or give a clue about the strategies of standardized medicinal plant remedy. The present study, therefore, aimed to evaluate the activities of *Cola anomala* water/ethanol pods extract on *Shigella flexneri* diarrhea model induced in rats.

## 2. Materials and Methods

### 2.1. Plant Material

In this study, the plant material was made up of pods from *Cola anomala* tree, which was identified at the Yaoundé National Herbarium, in comparison with the specimen referenced 50223HNC. The pods were collected from Batié (West Region, Cameroon, coordinates 9°25′17″N and 13°27′2″E) between March and April 2013 and were separated from the nut and, then, were cleaned, reduced into small pieces, shade dried, and crushed to get a powder. The powder was extracted with a water/ethanol mixture (V/V). For this, 1000 g of powder was macerated for 72 h in 5 L of a mixture of equal volume of water and ethanol at room temperature with occasional stirring and then filtered (Whatman filter paper No. 1). Furthermore, the residue was remacerated for 72 h in the same solvent (5 L) and filtered. The two obtained filtrates were pooled and concentrated in a rotary evaporator (BÜCHI Rota vapor R-124) at 65°C resulting in 175 g of a light brown hydroethanolic extract which was lyophilized and kept at +4°C until used. Extract solutions were prepared with distilled water, before oral administration to rats or for *in vitro* and *in vivo* experiments.

### 2.2. Experimental Animals

We used male and female healthy *Wistar* albino rats (90–200 g) obtained from the animal house of the Physiology and Phytopharmacology Laboratory of the University of Dschang. Animals were reared (1 rat/cage) at room temperature in clean metabolic cages with natural light/dark cycle and sufficient aeration. They had free access to water and standard rat diet, the composition of which for 1 kg was maize flour (60%), wheat flour (10%), fish (12%), soya bean (15%), and kernel cake (3%) enriched with the vitamin complex [16]. *In vivo* tests were carried out with respect to the European Union Guidelines on Animal Care (Council EEC 86/609) [17] adopted in Cameroon by the Institutional Committee of the Ministry of Scientific Research and Innovation.

### 2.3. Microbial Strains

The microbial strain used in this study was *Shigella flexneri*, a clinical strain obtained from the Centre Pasteur of Yaoundé, Cameroon. This microbial strain was isolated from local patients. Autoclaved (121°C, 15 min) Muller-Hinton agar medium (Diagnostic Liofilchem, Italy, Ref. 610033) was poured into sterile Petri dishes (4 mL/Petri dish, 3-4 mm depth) and allowed for solidification. Culture media were then inoculated by streaks with the microbial strain and then incubated in an oven (Memmert Model 700) at 37°C for 24 h. From this young culture, two colonies were isolated for the preparation of the inoculum.

### 2.4. *In Vitro* Antimicrobial Studies

#### 2.4.1. Impregnation of Disks

Sterile filter paper discs of 6 mm diameter and 1 mm thick were impregnated with 10 *μ*L of different solutions of *Cola anomala* pods water/ethanol extract (0.39, 0.78, 1.56, 3.12, 6.25, 12.50, 25.00, 50.00, 100.00, and 200.00 mg/mL) or with ciprofloxacin (Ryan PHARMA UK; 30 *μ*g/mL) and then oven-dried at 25°C for 24 h [2].

#### 2.4.2. Preparation of Bacterial Inocula

From the previously prepared *Shigella flexneri* culture, two colonies were collected and dissolved in 5 mL of sterile physiological saline. After homogenization, the opacity of the bacterial suspension in the tube was adjusted to 0.5 Mac Farland scale corresponding to 1.5 × 10^8^ CFU/mL [18]. The standard inoculum was obtained by dilution of this bacterial solution to 1/1000^th^ and thus to 1.5 × 10^5^ CFU/mL.

#### 2.4.3. Antimicrobial Susceptibility

All antibacterial tests were performed under strict aseptic conditions to ensure the quality of the results. Antimicrobial susceptibility screening of *Cola anomala* pods water/ethanol extract was performed by the disk diffusion method [19]. Culture media (Müller-Hinton Agar and *Salmonella Shigella* Agar, Titan Biotech Ltd. India 386) were inoculated with 500 *μ*L of newly prepared *Shigella flexneri* suspensions at 1.5 × 10^5^ CFU/mL. Having ensured that agar entire surface is covered, the excess microbial suspension was, however, eliminated with a sterile Pasteur pipette [20]. Disks previously impregnated with extract or with ciprofloxacin were then equidistantly deposited on the surface of the agar (5 per Petri dish), 15 minutes after inoculation. After 30-minute preincubation period under the hood, this preparation in Petri dishes was allowed for 24 h incubation at 37°C in an oven. After the incubation, diameters of inhibition were measured using an electronic caliper [21]. Each trial was done in triplicate [22, 23].

#### 2.4.4. Determination of the Minimum Inhibitory Concentration

The minimum inhibitory concentration (MIC) is the lowest concentration of an antibiotic which presents no visible microbial growth [24]. It was determined by agar dilution technique [2, 25]. All inhibition assays were conducted in triplicate. A solution set of extract with concentrations ranging from 50.00 to 0.05 mg/mL was prepared. Aliquot of each extract solution (2 mL) was added to 18 mL of presterilized molten Müller-Hinton agar or to 18 mL of *Salmonella Shigella* agar at 40°C leading to a final concentration ranging from 5.00 to 0.005 mg/mL. The medium in tubes was then allowed to solidify. The surfaces of the media received a 18 h *Shigella flexneri* inoculum, followed by 24 h incubation at 37°C. After this incubation, tubes were observed; the first in the series presenting no visible growth was considered as the MIC [2].

#### 2.4.5. Determination of Minimal Bactericidal Concentration

The minimum bactericidal concentration (MBC) of extracts on microbes was determined from the Petri dish previously considered as MIC. In fact, the agar surface of the Petri dish which showed no visible bacterial growth was scraped and streaked with an inoculation loop on the surface of sterile MH agar. Incubation was done at 37°C for 24 h. The lowest concentration with no bacterial colony (99.99% kill) was considered as the MBC of the extract on the tested strain [2].

### 2.5. *In Vivo* Antimicrobial Studies: *Shigella flexneri*-Induced Diarrhea in Rats

For shigellosis induction, *Shigella flexneri* inoculum was adjusted to an equivalence with the turbidity of point 4.0 of the Mac Farland scale, which approximately corresponds to 12 × 10^8^ CFU/mL.

Before the experiment, all animals were deparasitized by daily administration (6 : 00 am and 6 : 00 pm) of 5 mg/kg Azithromycin (Greenstone LLC Peapack NJ07977) for 3 consecutive days. After the deparasitization, the rats were individually isolated in metabolic cages and, with the exception of those in the normal control group (NC), received per os with the aid of a gavage tube, a 24 h inoculum of *Shigella flexneri*, adjusted to an equivalence with the turbidity of point 4.0 of the Mac Farland scale [26]. As soon as diarrheal stools appeared (24 h after *Shigella flexneri* administration), diarrheic rats were divided, according to their size, into five groups of five animals each:

- A diarrheal control group (DC) which received distilled water (10 mL/kg)
- A positive control group (CIP) which received ciprofloxacin (Ryan PHARMA UK) (2.5 mg/kg)
- Three test groups which orally received each *Cola anomala* extract at 25 (KEO25), 50 (KEO50), and 100 (KEO100) mg/kg, respectively

The animals received treatment for one week twice daily (6 : 00 am and 6 : 00 pm). Every day, stool weight and its *Shigella flexneri* density were recorded, as well as the body weight variation.

*Shigella flexneri* density was evaluated by collecting 0.5 g of fresh stool from each rat and dissolving it in 4.5 mL of sterile saline. From this stool solution, 250 *μ*L was further diluted in 9.750 mL of sterile saline. 50 *μ*L of this microbe suspension was finally taken and cultured on SS agar for 24 h at 37°C. *Shigella* density was then determined by direct *Shigella flexneri* colonies counting [2].

At the end of treatment, rats, including those in the normal control (NC) group, were sacrificed under anesthesia (Thiopentone, *ip* injection 0.1 mL/100 g bw) [27], and blood collected in EDTA tubes was used for blood cell count using a hematology analyzer (Golden Harvest Industries BC 2800 Hematology Analyzer). Colon was also collected. Part of it served in histological studies while homogenate from another portion was centrifuged at 10,000 rpm for 15 min. The supernatant was collected in Eppendorf tubes and used to determine PGE_2,_ IL-1*β*, and TNF-*α* levels using ELISA method and for spectrophotometric evaluation of NO level [28].

### 2.6. Data Analysis

The results were expressed as means ($\bar{\text{X}}$) affected by the standard errors of the mean (SEM): $\bar{\text{X}}\pm \text{SEM}$, and they were presented in the form of tables or figures. Control and test group means were compared by one-way analysis of variance (ANOVA) followed by Dunnett's test using GraphPath Prism 7, Version 7.04 software.

## 3. Results

### 3.1. Antimicrobial Susceptibility

*In vitro*, hydroethanolic extract of *Cola anomala* inhibited *Shigella flexneri* growth (Table 1). The most important inhibition was 20.09 ± 0.27 mm at 6.25 mg/mL. Ciprofloxacin, the reference drug, inhibited *Shigella flexneri* growth with a diameter of about 28.00 ± 0.30 mm at 0.03 mg/mL.

### 3.2. Minimal Inhibitory Concentration (MIC) and Minimum Bactericidal Concentration (MBC)

The water/ethanol extract of *Cola anomala* and ciprofloxacin *in vitro* inhibited *Shigella flexneri* growth (Table 2). MBC/MIC ratios of *Cola anomala* and ciprofloxacin were 1.5 and 2.0, respectively (Table 2).

### 3.3. *In Vivo* Activities of Extract of Cola Anomala Pot and Ciprofloxacin on *Shigella flexneri*-Induced Diarrhea in Rats

#### 3.3.1. Effect on Animal Behavior and Stool Appearance

**Figure 1:**
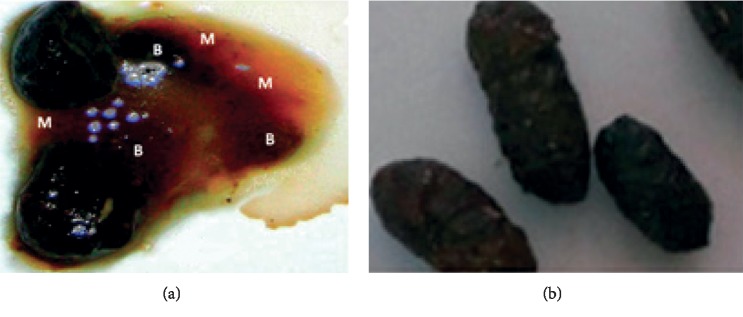
Stools appearance after diarrhea induction in rat ((a) diarrheic stool) and after 7 days of treatment with *C. anomala* pods water/ethanol extract or with ciprofloxacin ((b) normal stool). B: blood; M: mucus.

Four hours after *Shigella flexneri* administration, rats became calm and folded into a ball with erected furs, signs of abdominal cramps. The first diarrheal stools appeared 20 h after induction. These unmolded stools presented traces of blood and mucus (Figure 1). Treated diarrheal animals (with different extract doses or ciprofloxacin) gradually recovered mobility, with normal stools.

#### 3.3.2. Effect on *Shigella flexneri* Stool Density

**Figure 2:**
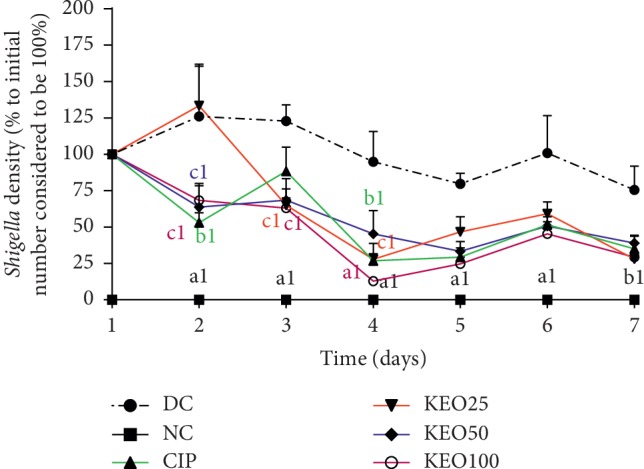
*Shigella flexneri* load in rat stools after one week of treatment with *C. anomala* pods water/ethanol extract at 25 (KEO25), 50 (KEO50), and 100 (KEO100) mg/kg or ciprofloxacin 2.5 mg/kg (CIP) (*n* = 5). Significant difference: a1 (*P* < 0.001), b1 (*P* < 0.01), and c1 (*P* < 0.05) compared to diarrheic control (DC).

In diarrheic control (DC) rats, *Shigella flexneri* load increased and was significantly (*P* < 0.001) high from the onset of diarrhea in the first to the third day (Figure 2). In treated animals, the number of bacteria significantly (*P* < 0.001) decreased from the first day of treatment (Figure 2). The stool *Shigella* density of rats treated with the extract of *C. anomala* pods at 25, 50, or 100 mg/kg (KEO25, KEO50, or KEO100) significantly decreased (*p* < 0.01) during the first to the seventh day of treatment. At day seven, bacterial loads reduced of about 28.01, 39.12, 29.41, and 35.10%, respectively, by KEO25, KEO50, KEO100, and ciprofloxacin.

#### 3.3.3. Weight Evolution of Rats

**Figure 3:**
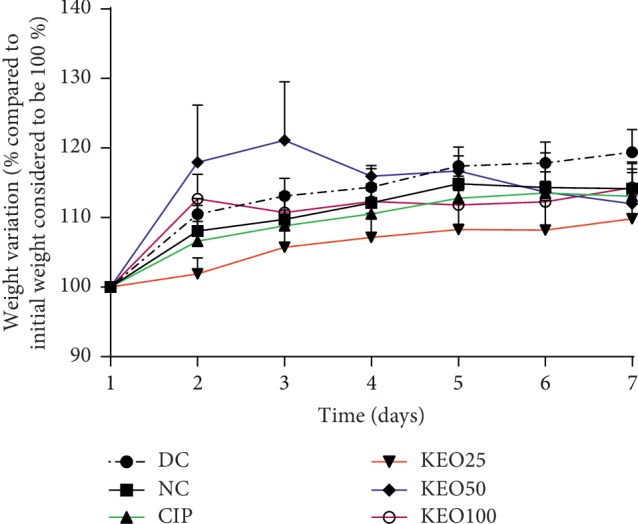
Mean body weight variation in *Shigella flexneri*-induced diarrhea rats during one-week administration of *C. anomala* water/ethanol extract at 25 (KEO25), 50 (KEO50), and 100 (KEO100) mg/kg or ciprofloxacin 2.5 mg/kg (CIP) (*n* = 5).

From the first to the seventh day of treatment, the body weight of normal (NC) or diarrheal (DC) rats treated with extract or ciprofloxacin showed no important change (Figure 3).

#### 3.3.4. Effects on Blood Cells Count

In diarrheal control (DC) rats, we observed a significant (*P* < 0.01) increase in white blood cells: 6.4 ± 0.2 × 10^9^ against 4.2 ± 0.2 × 10^9^/L for normal rats. Red blood cell (RBC), haemoglobin (Hb), hematocrit (HCT), platelets (Pc), mean corpuscular haemoglobin concentration (MCHC), mean globular haemoglobin (MGH), and mean globular volume (MGV) did not significantly change (Table 3).

#### 3.3.5. Effects on Intestine Morphology of *Shigella flexneri*-Induced Diarrhea Rats

**Figure 4:**
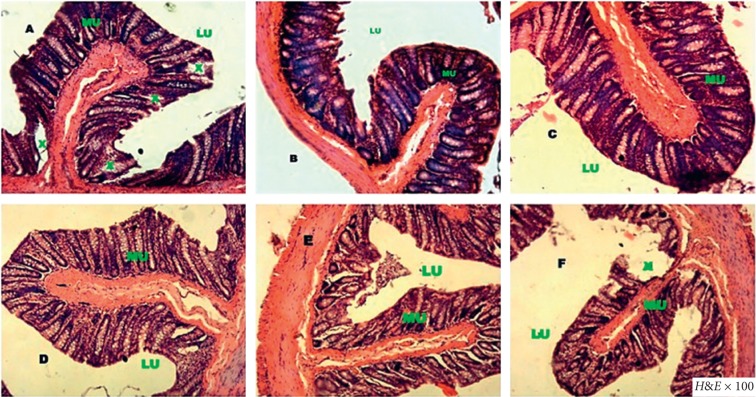
Histology of the colon in diarrheic control rats (a), normal rats (b), and diarrheic rats treated with ciprofloxacin 2.5 mg/kg (c) or with *C*. *Anomala* pods water/ethanol extract at 25 mg/kg (d), 50 mg/kg (e), and 100 mg/kg (f). MU: mucosa; X: alteration; LU: lumen.

Diarrheal rats' intestine epithelium (A) presented significant alteration with more mucus and total erosion of microvilli with straight and angular surface compared to normal rats (B). Treatment with ciprofloxacin (C) as well as hydroethanolic *C. anomala* extract at 25 (D), 50 (E), and 100 mg/kg (F) almost completely restored the epithelium structure (Figure 4).

#### 3.3.6. Effects on Some Biochemical Parameters of *Shigella flexneri*-Induced Diarrhea Rats

Biochemical parameters evaluated were nitric oxide (NO), prostaglandin *E*_2_ (PGE_2_), proinflammatory cytokines interleukin 1 beta (IL-1*β*), and tumor necrosis factor alpha (TNF-*α*).

**Figure 5:**
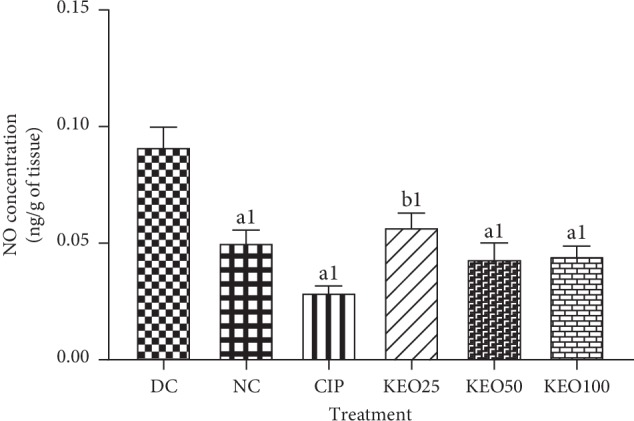
NO concentration in colon homogenate of *Shigella flexneri*-induced-diarrhea rats during one-week treatment with *C. anomala* pods water/ethanol extract at 25 (KEO25), 50 (KEO50), and 100 (KEO100) mg/kg or ciprofloxacin 2.5 mg/kg (CIP) (*n* = 5). Significant difference: a1 (*P* < 0.001) and b1 (*P* < 0.01) compared to diarrheal control (DC).

*(1) Effects on Shigella flexneri-Induced Diarrheic Rats NO Production in the Colon*. NO level in untreated diarrheal control (DC) rats was significantly (*P* < 0.001) high compared to normal animals (NC). Treatment with hydroethanolic *C. anomala* extract at 25 (KEO25), 50 (KEO50), and 100 (KEO100) mg/kg or with ciprofloxacin (2.5 mg/kg) dose dependently and significantly (*P* < 0.001) decreased NO concentration in colon homogenate (Figure 5).

**Figure 6:**
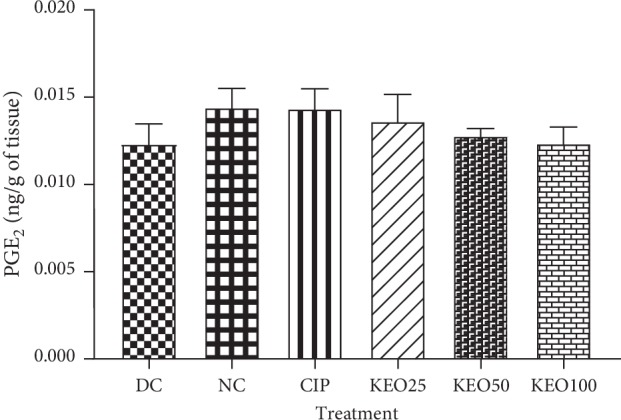
PGE_2_ concentration in colon homogenate of *Shigella flexneri*-induced diarrhea rats during one-week treatment with *C*. *anomala* pods water/ethanol extract at 25 (KEO25), 50 (KEO50), and 100 (KEO100) mg/kg or ciprofloxacin 2.5 mg/kg (CIP) (*n* = 5).

*(2) Effects on Shigella flexneri-Induced Diarrhea Rats PGE*_*2*_ *Production in the Colon*. *Shigella flexneri* infection was not found to be of great importance on PGE_2_ production in rat colon. PGE_2_ concentrations in various treated groups were not significantly different compared to those in normal or untreated groups (Figure 6).

**Figure 7:**
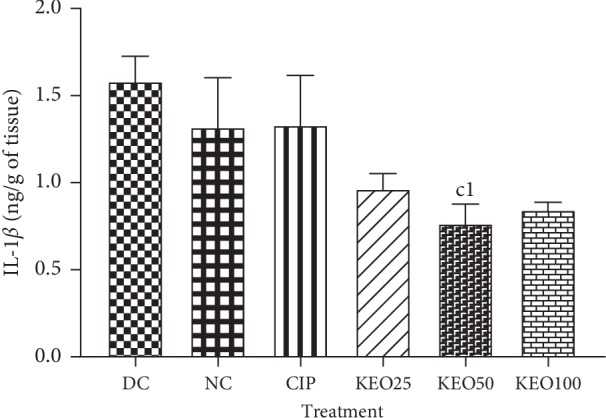
IL-1*β* concentration in colon homogenate of *Shigella flexneri*-induced diarrhea rats during one-week treatment with *C. anomala* pods water/ethanol extract at 25 (KEO25), 50 (KEO50), and 100 (KEO100) mg/kg or ciprofloxacin 2.5 mg/kg (CIP) (*n* = 5). Significant difference: c1: *P* < 0.05, compared to diarrheic control (DC).

*(3) Effects on Shigella flexneri-Induced Diarrhea Rats IL-1β Production in the Colon*. *Shigella flexneri* infection induced a slight increase of IL-1*β* in diarrheal untreated rats. Its concentration per gram of tissue was about 1.58 ng/g. *C*. *anomala* or ciprofloxacin treatment nonsignificantly decreased IL-1*β* colon production with a concentration of 0.96, 0.77, 0.84, and 1.33 ng/g of tissue, respectively, for the extract at 25 (KEO25), 50 (KEO50), and 100 (KEO100) mg/kg or ciprofloxacin 2.5 mg/kg (Figure 7).

**Figure 8:**
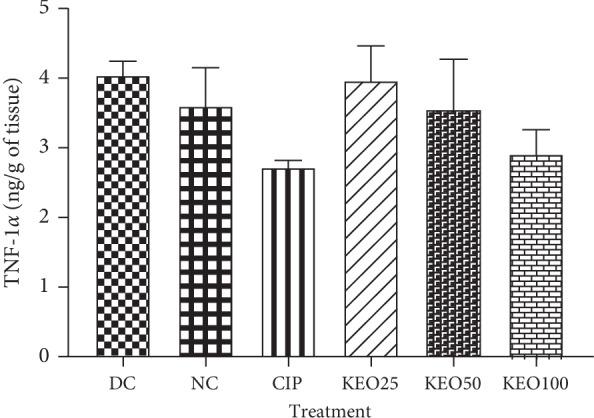
TNF-*α* concentration in colon homogenate of *Shigella flexneri*-induced diarrhea rats during one-week treatment with *C. anomala* pods water/ethanol extract at 25 (KEO25), 50 (KEO50), and 100 (KEO100) mg/kg or ciprofloxacin 2.5 mg/kg (CIP) (*n* = 5).

*(4) Effects on Shigella flexneri-Induced Diarrhea Rats TNF-α Production in the Colon. Shigella flexneri* infection brought a nonsignificant increase of TNF-*α* colon production, about 4.04 ng/g of tissue in diarrheal untreated rats against 3.60 ng/g of tissue in normal control. *C. anomala* water/ethanol extract treatment slightly decreased TNF-*α* concentration which was of about 3.97, 3.55, and 2.91, respectively, in rats receiving *C. anomala* water/ethanol extract at 25 (KEO25), 50 (KEO50), and 100 (KEO100) mg/kg (Figure 8).

## 4. Discussion

This work was carried out to verify the scientific claim of the use of *Cola anomala* in traditional medicine against diarrheal diseases. *C. anomala* water/ethanol extract antidiarrheal activities were investigated, *in vitro* on *Shigella flexneri* growth inhibition and *in vivo* on *Shigella flexneri* diarrhea model in rats.

The extract *in vitro* inhibited *S. flexneri* growth with an MBC/MIC ratio ≤4, thus indicating bactericidal effect of the extract [26]. *In vivo*, the extract protected infected rats against the deleterious effects of *S*. *flexneri* infection. *S*. *flexneri* is responsible for acute bloody diarrhea through invasion and destruction of the colonic epithelium. This sometimes leads to fever and stomach cramps or to the formation of microulcers and inflammatory exudates and causes inflammatory cells and blood or mucous to appear in stools [7]. Stomach cramps in shigellosis may results from Shiga-toxin action [29]. In this study, symptoms of shigellosis in infected treated rats significantly reduced with respect to the decrease of *S*. *flexneri* stools load. This confirms the *in vitro* and *vivo* antimicrobial activity of the extract which showed to have bactericidal activity. The reduction of stools bacterial load in treated groups was further associated with a decrease of NO, IL-1*β*, and TNF-*α* colon production. This treatment had no significant effect on PGE_2_ level.

Bacterial antigen, lipopolysaccharides (LPS), is an endotoxin in cell walls of Gram-negative bacteria. It is responsible for the inflammation of colon mucosa and can initiate a great number of hemodynamic and metabolic changes [30]. LPS is responsible for the activation of macrophages resulting in the secretion of biologically active substances, including prostaglandins, an arachidonic acid metabolite; nitric oxide; and cytokines [30]. Furthermore, LPS is thought to increase inducible NO synthase (iNOS) expression with NO production in muscularis resident macrophages. Excess NO level stimulates gastrointestinal tract secretion by the increase of intracellular cAMP and cGMP levels [30]. In this study, *C. anomala* water/ethanol extract reduced NO concentration in colon homogenate. This activity probably may be the result of the inhibition of iNOS activity or of the inhibition of its production. LPS also can alter the normal influx/efflux ratio and particularly reduce the lumen-to-blood fluid influx. However, this extract showed no significant effect on PGE_2_ concentration indicating that the extract may not have any activity on cyclooxygenase 2 (COX-2).

The sequence of events in *S. flexneri* infection starts with the invasion of the bacteria by translocation through M cells from the lumen of the colon into the submucosa; phagocytosis of *Shigella* by macrophages; escape of bacterial vacuolar from the phagosome into the cytoplasm of the macrophage; production of invasion plasmid antigen B (IpaB); the distribution of IpaB through the macrophage's cytoplasm; binding and activation of interleukin-1*β* converting enzyme (ICE); and induction of apoptosis and cleavage of IL-1*β* [31]. *C*. *anomala* and ciprofloxacin in treated animals slightly reduced IL-1*β* production in the colon. This extract might contain bioactive substances which have direct effect on *S. flexneri* destruction or which may inhibit one of the infections steps previously described. Similar results were obtained by Kamgang et al. with *Euphorbia scordifolia* [19] or by Noubissi et al. with *Crinum jagus* [2]. In fact, phytochemical studies have revealed the presence of alkaloids and phenolic compounds in *Cola* sp. [32], where phenolic compounds are shown to be responsible for bacteria plasma membrane rupture, thus increasing its permeability [33].

Early cytokine production (TNF-*α* and IL-1*β*) correlated with weight loss and histopathological affection of colon mucosa is implicated in the pathogenesis and *S. flexneri* infection outcomes [34]. *C. anomala* and ciprofloxacin treatment protected diarrheic rats from excessive weight loss, colon mucosa damage, and, furthermore, slightly decreased TNF-*α* production. This may result from a direct bactericidal effect on luminal *S. flexneri* confirmed by decreased *S. flexneri* count in stools. Ciprofloxacin is a synthetic bactericidal antibiotic which belongs to fluoroquinolone family and is recognized for its great anti-*S. flexneri* activity [29]. It inhibits DNA gyrase activity and, thus, prevents the supercoiling of the bacterial chromosome [29]. *C*. *anomala* may contain potential bioactive molecules acting via the same mechanism.

## 5. Conclusion

*In vivo*, *Cola anomala* extract inhibited bacterial growth and showed to be bactericidal. In infected treated rats, it decreased bacterial load and protected them against weight loss and colon mucosal damage. Furthermore, the extract decreased PGE_2_, IL1*β*, TNF-*α*, and NO production. These results support the use of the plant in traditional medicine in the treatment of gastrointestinal ailments and could therefore be a potential candidate for the production of improved forms of traditional medicines, cheap and available for local population for effective treatment of shigellosis. In future studies, extract activities will be evaluated on other models of experimentally induced diarrhea. Also, detailed phytochemical profiles of the extract will be explored so as to elucidate the major compounds underlying the observed activities.

## Figures and Tables

**Table 1:** *Shigella flexneri* sensitivity to *Cola anomala* hydroethanolic extract and ciprofloxacin.

Treatments	Concentrations (mg/mL)	Inhibition diameters (mm)
*Cola anomala*	0.39	19.27 ± 0.72
	0.78	14.80 ± 0.24
	1.56	14.76 ± 4.05
	3.13	19.34 ± 0.87
	6.25	20.09 ± 0.27
	12.50	17.02 ± 0.36
	25.00	15.88 ± 1.62
	50.00	16.70 ± 0.77
	100.00	17.35 ± 0.68
	200.00	09.08 ± 0.50

*Ciprofloxacin*	0.03	28.00 ± 0.30

**Table 2:** Minimum bactericidal concentration (MBC) and minimum inhibitory concentration (MIC) of *Cola anomala* water/ethanol extract and ciprofloxacin on *Shigella flexneri*.

Inhibition parameters	*Cola anomala*	Ciprofloxacin
Minimum bactericidal concentration (mg/mL)	3.00	4.0 × 10^−3^
Minimum inhibitory concentration (mg/mL)	2.00	2.0 × 10^−3^
MBC/MIC ratio	1.50	2.00

**Table 3:** Blood cell count in *Shigella flexneri*-infected rats after treatment with *C*. *anomala* water/ethanol extract and ciprofloxacin.

	DC	NC	CIP	KEO25	KEO50	KEO100
WBC × 10^9^/L	6.40 ± 0.20	4.20 ± 0.20^b1^	5.00 ± 0.50	6.60 ± 0.50^b^	4.80 ± 0.30	5.20 ± 0.30
RBC × 10^12^/L	6.62 ± 0.39	6.03 ± 0.32	6.57 ± 0.20	6.45 ± 0.49	6.34 ± 0.33	6.28 ± 0.25
Hb (g/dL)	12.90 ± 0.60	12.00 ± 0.50	13.00 ± 0.20	12.80 ± 0.50	12.80 ± 0.30	12.70 ± 0.20
HCT (%)	39.90 ± 2.10	36.10 ± 1.60	38.10 ± 1.50	37.80 ± 2.70	35.80 ± 1.30	35.60 ± 1.30
Pc × 10^9^/L	685.20 ± 45.70	760.80 ± 42.20	742.40 ± 20.60	708.00 ± 47.40	687.40 ± 28.80	702.20 ± 44.60
MGV (fL)	57.50 ± 1.50	59.40 ± 0.70	57.40 ± 0.60	57.00 ± 0.70	55.70 ± 1.10	57.20 ± 0.30
MGH (*ρ*g)	18.70 ± 1.10	21.20 ± 0.80	20.70 ± 0.70	19.80 ± 1.00	18.20 ± 1.00	20.10 ± 1.00
MCHC (g/dL)	35.10 ± 1.00	31.80 ± 1.40	35.00 ± 1.40	34.00 ± 1.40	32.70 ± 1.20	34.20 ± 1.70

KEO25, KEO50, and KEO100: *C*. *anomala* water/ethanol extract at 25, 50, and 100 mg/kg; CIP: ciprofloxacin 2.5 mg/kg; NC: normal control; DC: diarrheic control; WBC: white blood cell; Hb: haemoglobin; HTC: hematocrit; RBC: red blood cell; Pc: platelets; MGV: mean globular volume; MGH: mean globular haemoglobin; MCHC: mean corpuscular haemoglobin concentration (*n* = 5). Significant difference: b1: *P* < 0.01, compared to diarrheic control (DC); b: *P* < 0.01, compared to normal control (NC).

## Data Availability

The data used to support the findings of this study are available from the corresponding author upon request.
